# Supplementary material for: Modeling glioblastoma heterogeneity as a dynamic network of cell states
Source: Mol Syst Biol. 2021 Sep 16;17(9):e10105. doi: 10.15252/msb.202010105 (PMC8444284; doi:10.15252/msb.202010105)
Supplement: Supplementary file 5 — Source Data for Figure 3 [file MSB-17-e10105-s001.zip › Figure3A_sourcedata/GSEA_3065/nosigdb_state6.GseaPreranked.1622718443993/ANASTASSIOU_MULTICANCER_INVASIVENESS_PMID_22208948.html]

Details for gene set ANASTASSIOU\_MULTICANCER\_INVASIVENESS\_PMID\_22208948[GSEA]

|  || Dataset | state6 |
| Phenotype | NoPhenotypeAvailable |
| Upregulated in class | na\_neg |
| GeneSet | ANASTASSIOU\_MULTICANCER\_INVASIVENESS\_PMID\_22208948 |
| Enrichment Score (ES) | -0.6871666 |
| Normalized Enrichment Score (NES) | -2.1084218 |
| Nominal p-value | 0.0 |
| FDR q-value | 0.0 |
| FWER p-Value | 0.0 |
Table: GSEA Results Summary

  

Fig 1: Enrichment plot: ANASTASSIOU\_MULTICANCER\_INVASIVENESS\_PMID\_22208948      
 Profile of the Running ES Score & Positions of GeneSet Members on the Rank Ordered List

  

| PROBE | GENE SYMBOL | GENE\_TITLE | RANK IN GENE LIST | RANK METRIC SCORE | RUNNING ES | CORE ENRICHMENT || 1 | RAB31 |  |  | 23 | 0.284 | 0.0789 | No |
| 2 | NUAK1 |  |  | 1290 | 0.072 | -0.0448 | No |
| 3 | TMEM158 |  |  | 1598 | 0.061 | -0.0625 | No |
| 4 | FN1 |  |  | 2839 | 0.026 | -0.1965 | No |
| 5 | COPZ2 |  |  | 3660 | 0.008 | -0.2878 | No |
| 6 | AEBP1 |  |  | 4222 | -0.004 | -0.3507 | No |
| 7 | TIMP3 |  |  | 5017 | -0.019 | -0.4359 | No |
| 8 | PLAU |  |  | 5312 | -0.025 | -0.4623 | No |
| 9 | MMP11 |  |  | 6442 | -0.048 | -0.5772 | No |
| 10 | MMP2 |  |  | 7016 | -0.065 | -0.6237 | No |
| 11 | PRRX1 |  |  | 7482 | -0.081 | -0.6536 | No |
| 12 | FBN1 |  |  | 7777 | -0.094 | -0.6601 | Yes |
| 13 | CDH11 |  |  | 7885 | -0.101 | -0.6435 | Yes |
| 14 | COL6A2 |  |  | 8253 | -0.126 | -0.6491 | Yes |
| 15 | SPARC |  |  | 8303 | -0.132 | -0.6166 | Yes |
| 16 | COL5A1 |  |  | 8428 | -0.150 | -0.5878 | Yes |
| 17 | TNFAIP6 |  |  | 8434 | -0.150 | -0.5453 | Yes |
| 18 | THY1 |  |  | 8454 | -0.153 | -0.5036 | Yes |
| 19 | COL5A2 |  |  | 8514 | -0.165 | -0.4631 | Yes |
| 20 | COL11A1 |  |  | 8518 | -0.165 | -0.4160 | Yes |
| 21 | ADAM12 |  |  | 8526 | -0.168 | -0.3686 | Yes |
| 22 | NTM |  |  | 8612 | -0.196 | -0.3219 | Yes |
| 23 | PCOLCE |  |  | 8639 | -0.209 | -0.2650 | Yes |
| 24 | VCAN |  |  | 8664 | -0.219 | -0.2050 | Yes |
| 25 | LOXL2 |  |  | 8687 | -0.232 | -0.1408 | Yes |
| 26 | POSTN |  |  | 8795 | -0.533 | 0.0001 | Yes |
Table: GSEA details [plain text format]

  

Fig 2: ANASTASSIOU\_MULTICANCER\_INVASIVENESS\_PMID\_22208948: Random ES distribution      
 Gene set null distribution of ES for **ANASTASSIOU\_MULTICANCER\_INVASIVENESS\_PMID\_22208948**
